# New Anticancer Drugs: Reliably Assessing “Value” While Addressing High Prices

**DOI:** 10.3390/curroncol31050184

**Published:** 2024-04-28

**Authors:** David J. Stewart, John-Peter Bradford, Sandeep Sehdev, Tim Ramsay, Vishal Navani, Nigel S. B. Rawson, Di Maria Jiang, Joanna Gotfrit, Paul Wheatley-Price, Geoffrey Liu, Alan Kaplan, Silvana Spadafora, Shaun G. Goodman, Rebecca A. C. Auer, Gerald Batist

**Affiliations:** 1 Division of Medical Oncology, University of Ottawa, 501 Smyth Road, Ottawa, ON K1H 8L6, Canadajgotfrit@toh.ca (J.G.); pwheatleyprice@toh.ca (P.W.-P.); 2 Ottawa Hospital Research Institute, Ottawa, ON K1H 8L6, Canada; tramsay@ohri.ca (T.R.); rauer@ohri.ca (R.A.C.A.); 3 Life Saving Therapies Network, Ottawa, ON K1H 5E6, Canada; jp@bradski.net (J.-P.B.); gerald.batist@mcgill.ca (G.B.); 4 Division of Medical Oncology, University of Calgary, Calgary, AB T2N 1N4, Canada; vishal.navani@albertahealthservices.ca; 5 Canadian Health Policy Institute, Toronto, ON M5V 0A4, Canada; eastlakerg@gmail.com; 6 Macdonald-Laurier Institute, Ottawa, ON K1N 7Z2, Canada; 7 University of Toronto, Toronto, ON M5S 3H2, Canada; di.jiang@uhn.ca (D.M.J.); geoffrey.liu@uhn.ca (G.L.); for4kids@gmail.com (A.K.); goodmans@chrc.net (S.G.G.); 8 Princess Margaret Cancer Center, Toronto, ON M5G 2M9, Canada; 9 Family Physicians Airway Group of Canada, Markham, ON L3R 9X9, Canada; 10 Algoma District Cancer Program, Sault Ste Marie, ON P6B 0A8, Canada; spadaforas@sah.on.ca; 11 St. Michael’s Hospital, Unity Health Toronto, and Peter Munk Cardiac Centre, University Health Network, Toronto, ON M5B 1W8, Canada; 12 Department of Surgery, University of Ottawa, 501 Smyth Road, Ottawa, ON K1H 8L6, Canada; 13 Centre for Translational Research, Jewish General Hospital, McGill University, Montreal, QC H3T 1E2, Canada

**Keywords:** drug costs, drug value, ICER, QALY

## Abstract

Countries face challenges in paying for new drugs. High prices are driven in part by exploding drug development costs, which, in turn, are driven by essential but excessive regulation. Burdensome regulation also delays drug development, and this can translate into thousands of life-years lost. We need system-wide reform that will enable less expensive, faster drug development. The speed with which COVID-19 vaccines and AIDS therapies were developed indicates this is possible if governments prioritize it. Countries also differ in how they value drugs, and generally, those willing to pay more have better, faster access. Canada is used as an example to illustrate how “incremental cost-effectiveness ratios” (ICERs) based on measures such as gains in “quality-adjusted life-years” (QALYs) may be used to determine a drug’s value but are often problematic, imprecise assessments. Generally, ICER/QALY estimates inadequately consider the impact of patient crossover or long post-progression survival, therapy benefits in distinct subpopulations, positive impacts of the therapy on other healthcare or societal costs, how much governments willingly might pay for other things, etc. Furthermore, a QALY value should be higher for a lethal or uncommon disease than for a common, nonlethal disease. Compared to international comparators, Canada is particularly ineffective in initiating public funding for essential new medications. Addressing these disparities demands urgent reform.

## 1. Introduction

The objectives of this manuscript are to address why new anticancer drugs are so expensive and why current approaches to deciding a drug’s value can be problematic. This is a perspective analysis from a diverse group of co-authors from across Canada. The group includes medical oncologists, other physicians, a patient advocate/cancer survivor/psychopharmacologist, a pharmacoepidemiologist/pharmaceutical policy researcher and a biostatistician/epidemiologist.

Issues analyzed in this paper include major driving factors behind high drug prices, how countries may assess a new drug’s value in deciding how much they are willing to pay for it, Canada’s approach to deciding the value of a new drug, and some key potential issues in assessing a therapy’s value.

Canada is used as an example to illustrate both potential limitations in health technology assessments (HTAs) as well as how HTA approaches may adversely impact access to effective new cancer therapies. Recommendations are included on steps that could be taken to bring down drug prices, on patient benefits that might be considered in assessing a drug’s value, and on factors that Canada (as an example) should consider in deciding how much some of these incremental benefits might be worth.

## 2. The Link between High Drug Prices and High Drug Development Costs

***High prices for anticancer drugs:*** Countries differ in how much they are willing to pay for a new drug [1]. New therapies are expensive, and prices are rising rapidly [2,3]. For example, in the United States in 2012, the average cost to treat a patient with a new anticancer agent was approximately USD 89,000 per year [2]. By 2016–17, the amount had almost doubled to USD 174,000 [3].

***Factors driving high drug prices:*** As recently reviewed [4], several factors are driving high therapy prices. These include “personalization” of therapies (i.e., only using the therapy in a subpopulation of patients who have characteristics that potentially predict the therapy will be effective). This results in a relatively small market size [5]. If only a small proportion of the population has the characteristics known to be required for therapy efficacy, then relatively few patients will be treated. Hence, more may be charged for each patient treated so that drug development costs can be recouped.

There are also several factors that distort market forces. These include “comparative pricing” and “charging what the market will bear” (since one company is successful in setting a record price for a new drug, another company selects a similar high price for their unrelated new drug) [3], marketing approaches by drug companies [4], the presence of oligopolies (with relatively few companies responsible for most new drugs) [2,6], factors impairing competition between drugs [2,4], misperceptions about a drug’s efficacy [2,6], lobbying by pharmaceutical companies [7], factors that limit the impact of generic drugs [2], and restrictions on drug imports [4,8].

Another factor is that in 2003, the American government passed The Medicare Prescription Drug, Improvement and Modernization Act [2]. This law specifically blocked Medicare from negotiating with a manufacturer on drug prices. Consequently, US Medicare (but not other branches of the US government) had to accept the price proposed by the drug manufacturer. This is a key reason that American drug prices are the highest in the world [4,6].

If some purchasers of a product are willing to pay a very high price, then this may set the price bar higher, and a lower initial price may bring the overall global price bar down. It has been proposed that high unregulated initial American drug prices tend to increase worldwide prices [4]. If this is so, then recent initiatives to bring down US drug prices also might help reduce drug prices elsewhere, although they also could potentially slow access to new therapies by reducing the incentive to invest in new drug development [9,10].

***High drug development costs:*** A particularly important factor is that the cost of bringing a new drug from discovery to approval is rising much faster than inflation [11,12]. While estimates of drug development costs vary, it is clear that they are very high, particularly for anticancer drugs [13]. It cost an average of USD 4M to bring a new anticancer drug from discovery to marketing in the 1960s [14]. By the 1980s, this had increased to USD 230M [14], and by 2013, the estimate had risen to USD 2.9B [11]. These drug development costs eventually must be recovered from sales of approved drugs if investment in new drug development is to continue [4].

While bringing down drug development costs would not be sufficient to bring down drug prices, it is an important component [4]. Increasing personalization of therapy will continue to create upward pressure on drug prices since personalization means a smaller market size from which drug development, production and marketing costs may be recouped [5]. Irrespective of any other factors driving high drug prices, if governments take actions that bring revenues from drug sales down to levels lower than what it costs to develop these drugs, then private investment in drug development would be expected to decrease substantially or stop [4].

Since drug development costs have been rising much faster than inflation [11,12] and since there has been no concerted effort to address this, we believe that tackling high drug development costs constitutes an important opportunity that ultimately could help make a major difference in drug prices. The other factors driving high prices would still need to be addressed, but governments and companies would have more room to maneuver if drug development costs were much lower [4]. Regulation is highly important, and the objective would not be to weaken regulation but instead to make it more cost-effective.

The essential but excessively burdensome clinical research regulation that contributes substantially to high drug development costs [4,15] is “harmonized” internationally [16]. This harmonization has the advantage that it means clinical research data generated in one country can be used for approval of the drug in a different country, but it has the disadvantage that major changes cannot be made to clinical research regulation without international agreement [4].

With respect to individual regulatory factors driving high drug development costs, it can take years [17] for the costly preclinical toxicology assessments that may be required by regulators before clinical trials can be launched but that add little value [15]. Preclinical toxicology assessments generally predict the obvious (e.g., the drug will cause neutropenia) or miss toxicities ultimately found to be important in humans or predict toxicities that end up not being a problem. There are few examples of them giving a vital forewarning about a toxicity that is both unexpected and important [4].

The greatest potential value of preclinical toxicology assessments is to determine the “rodent LD10” (i.e., the drug dose that is a lethal dose, or LD, for 10% of rodents). It generally is safe to initiate human clinical trials with 10% of the rodent LD10 [18,19]. This can be achieved at a relatively low cost over just a few months.

If patients are to be selected for a trial based on a biomarker, then a biomarker test that is certified according to the Clinical Laboratory Improvement Amendments (CLIA) (or equivalent) must first be developed. The requirement for CLIA certification of a screening test can delay trial initiation by a year or more and can cost USD 100K before it is even known whether the test might predict drug efficacy [20]. A more rational strategy would be to initially use a much less expensive research laboratory test and only invest time and money in perfecting and certifying the test if the preliminary data suggested that it would be useful [4,20,21].

Multiple additional bottlenecks and barriers markedly slow clinical trial activation while increasing costs and adding little value [22,23]. There is an excessively high administrative burden interacting with Institutional Review Boards (IRBs) [24], and IRB approval of a study can take far too long [25]. The entire IRB approach needs a very major overhaul [26].

In addition to trial activation requiring IRB approval, most study amendments also require IRB approval, even if changes are minor and unlikely to negatively impact patient safety or study scientific conclusions. Approval of amendments can take several months or more and in some cases can cost hundreds of thousands of dollars [27].

Trial eligibility criteria are irrationally restrictive [28,29], with fewer than 5% of North American adults with cancer able to participate in a clinical trial [30], despite a majority being willing to participate if given the option [31]. Until the late 1990s, a trial’s Principal Investigator (PI) could grant an exception to the eligibility criteria and permit entry of a patient on a trial if there was no good clinical or scientific reason to exclude them. This generally is no longer allowed, but it makes little sense to not allow it if the patient’s participation would be unlikely to risk their safety or the conclusions of the trial [29].

Overall, the complexity of cancer clinical trials is rising rapidly [32]. For example, by 2011–2015, there was an 88% increase in the number of data points that were required per patient on a trial compared to 2001–2005 [33]. Typically, a large amount of expensive data must be collected during a clinical trial, but much of this information may add little value and may never be needed or used [34,35].

Involvement of a Clinical Research Organization in a trial can substantially increase the amount of expensive but unimportant data collected [4,34]. With the large amount of unimportant but “required” data that must be collected, the overwhelming majority of random FDA audits identify at least some deficiencies [36]. It is almost impossible to completely avoid errors when required to collect an unnecessarily huge amount of unhelpful data.

Increasing trial complexity has meant an increase in research trial staffing requirements [37] and in mandated procedures and patient visits [38]. Increasingly complex privacy restrictions have also increased clinical research costs and barriers [39,40]. The average cost to a sponsor for a patient on a phase III trial rose from around USD 26,000 in 2006 [15] to USD 41,413 by 2015–2017 and was as much as USD 75,000–90,000 in some studies [41,42]. Inflation [43] would have accounted for only USD 4953 of that USD 15,400 average cost increase.

Patient safety has been the major rationale given for this increased regulatory stringency [15]. However, as trial costs and complexity have increased, there has been negligible meaningful impact on patient safety, but at the very high cost of an estimated USD 2.7M per life-year saved by this increased regulatory burden [15]. Overall, clinical research regulation is both essential and well-intentioned, but it currently is not cost-effective [15]. The approaches used are not evidence-based and result in clinical research costs that are much too high. This inevitably contributes substantially to high drug prices [4].

Expensive new medications are usually paid for by governments or insurance companies. We anticipate that the number of new drugs developed would plummet if patients had to pay full price out-of-pocket since few could afford it. Hence, there would be insufficient sales to generate the profits needed to drive the research innovation needed for progress.

However, drug development costs are a direct function of regulatory processes and requirements [44]. The more cumbersome and complex, the longer it takes and the more expensive it is to develop drugs. Hence, an alternative scenario would be the evolution of a simpler regulatory environment, which would permit rapid, less expensive drug development. The governments that bear the burden of paying high drug prices have it within their power to lower these prices by rectifying markedly cost-ineffective clinical research regulations [4].

There have been attempts to improve the current situation [45,46,47,48,49,50], but we need to go much further. As briefly summarized in Table 1, there are several feasible steps that could be taken to markedly cut drug development costs without unreasonably jeopardizing safety or data integrity. These recommendations are derived from a Life-Saving Therapies Network-modified multi-round Delphi study involving 70 Canadian, European and American patient advocates and experts in drug development, ethics, oncology, and regulatory and legal processes [21] and from related prior publications [4,15,21,44,51,52].

The cost of enforcing regulations is also high. For example, the 2018 budget for the European Medicines Agency (EMA) was EUR 337,761,000 [56] (USD 368,159,500). In the US, several government agencies directly or indirectly impact new drug development [15]. The two with the most direct impact are the Food and Drug Administration (FDA) (2022 budget of USD 6.5B [57]) and the Office of Human Research Protection (OHRP). These costs indirectly impact drug prices by leading to increased fees charged by governments.

Regulatory rules can be applied rigidly or flexibly [15]. We would anticipate that applying effective flexible regulation generally would be more costly and difficult since there would be a need for greater oversight, judgment, background knowledge and societal consensus on regulation objectives. With rigid regulation, the regulator needs only determine whether the regulated company or investigator met specific criteria based on the current interpretation of the letter of the law. With flexible regulation, however, the regulator must assess nuances around whether the regulated person or entity complied with the underlying intent and principles of the law [15].

If anything goes wrong, regulators can defend themselves more easily if they apply regulations rigidly, while with more flexible regulation, their actions and judgments are more open to criticism. This favors “regulatory fundamentalism”: applying the current (and often shifting) interpretation of the exact wording of the law rigidly rather than applying the original intent of the law [15].

Rigid “zero tolerance” regulation may be easier for regulators, but it is generally more expensive and limiting for those who are being regulated [15]. Examples from education and legal systems demonstrate that it carries a high risk of unintended negative consequences [58,59,60]. Excessively rigid regulation of clinical research contributes to rapidly rising costs for approval and delayed availability of new therapies [4,15]. This translates into a heavy, dual societal cost of rapidly rising drug prices plus up to tens of thousands of life-years lost due to delayed access to effective new therapies [4,61].

Regulatory bodies also differ in their priorities and objectives. For example, the US FDA attempts to make effective new agents rapidly available to patients through “breakthrough drug” or “accelerated approval” designation [46] and similar approaches. In contrast, the US OHRP reportedly [26] attaches little importance to making essential new therapies available to patients quickly.

While regulation is essential, rigid regulation is costly to society [4,15]. The previous experience with the development of AIDS treatments [62] and more recently COVID-19 vaccines [63] clearly demonstrates that more flexible regulation and a focus on rapid progress can safely promote faster access to effective new therapies when governments decide to take action. However, so far, they have not chosen to do this for cancer.

For both AIDS and COVID-19, governments were pressured to act quickly. AIDS activists forced governments to make the changes needed for faster access to AIDS therapies [64]. For COVID-19, public health pressures were crucial to the faster development of effective vaccines. Prior to COVID-19, the average time required to develop a new vaccine was more than 10 years [65], and the fastest development was 4 years [66]. We believe that we now need similar activism and pressure to promote much faster, less expensive development of drugs for all lethal and severely debilitating diseases.

Several advocacy groups promote improved therapy access for individual diseases. In Canada, many of these groups are now working together through the CanCertainty Coalition and similar initiatives to “improve the affordability and accessibility of cancer treatment” [67]. We applaud the work of Canada’s advocacy groups.

In clinical research, regulation to ensure safety is essential [4]. However, for lethal diseases, the disease itself is by far the biggest danger to the patient. Patients with metastatic cancer are much more willing to face the risk of high toxicity in return for a smaller potential for benefit than are unafflicted members of the population [68]. For nonlethal diseases, a safety-centered approach is of prime importance. For lethal diseases, we need a “progress-centered” approach [4,44]. By slowing progress and markedly escalating clinical research costs, the current safety-centered approaches for lethal diseases are not cost-effective [15] or evidence-based, and they translate into a loss of huge numbers of life years [61] and rapidly rising drug prices [4]. The current approach needs to change [4,15,21,26,51,52,61].

The approaches we advocate would mean the availability of less data at the time of drug approval. We anticipate that this would increase risks, but the rapid availability of effective new therapies is highly important to patients with lethal diseases [61]. To ensure both the safety and efficacy of the therapy, this would make post-marketing surveillance even more important than it currently is [52]. We believe that the rapid migration to electronic medical record systems means that artificial intelligence could potentially prove to be a very valuable tool in generating real-world evidence as a cornerstone of this post-marketing surveillance.

## 3. Controlling Drug Prices

***Government price controls:*** Governments could choose to reduce drug prices through price controls. These might work in the short term, but price controls usually eventually fail since they result in commodity shortages [69]. If producers can no longer recoup costs, they stop producing, or they shift their products to other, more profitable markets, or they preferentially divert investment dollars to different sectors. For example, in response to the American introduction of the Inflation Reduction Act, the pharmaceutical company Pfizer has announced that it will be shifting its drug development priorities from oral small molecular therapies to biologic agents [10]. For new drugs, government price controls render a given jurisdiction a lower priority for manufacturers, resulting in delays in regulatory applications and in approvals for marketing and reimbursement. Substantially reducing prices in one jurisdiction can impact a company’s global business, and this affects how a company is likely to approach marketing decisions in a given country.

***Government willingness to pay, quality-adjusted life years and incremental cost-effectiveness ratios:*** Several factors may be considered in assigning value to healthcare interventions [70]. The impact of the therapy on life expectancy and quality of life, the cost of the new therapy, and the burden on the system of integrating a new drug are among the factors that are considered when a government decides if it will pay for a new therapy [71]. Stakeholders differ in their perspectives of what constitutes value [72,73,74,75]. Patients, their loved ones, and their physicians want access to therapies that improve cancer symptoms, prolong life, and offer hope. Governments also want affordability and “value”. Different governments may use different strategies in deciding how to approach high drug costs, and various “value calculators” have been devised [76,77]. Each of the current methods of calculating value has limitations [76,78,79,80,81].

One approach that governments commonly take is to develop an “incremental cost-effectiveness ratio” (ICER) for the new therapy that compares it to other available therapies [76,82]. There are various approaches to quantifying benefits. Once determined, the quantity of benefit with a standard therapy is then subtracted from the quantity of benefit with a new therapy to determine the quantity of benefit gained with the new therapy. To define the ICER for the new therapy, this gain in benefit is then divided by the added costs for the new therapy.

Governments may use the ICER to determine whether to fund the drug or how much to pay for it. For example, they may specify that they are willing to pay up to a given amount per “quality-adjusted life-year” (QALY) gained [82]. A QALY is calculated by multiplying the number of life-years gained by a “utility” value from 0 to 1, where dead is 0 and perfect health is 1 [83]. However, QALYs may be based on shaky assumptions, and they poorly reflect what many in the population deem to be important [84,85,86,87]. QALYs also typically do not consider the potential for an effective new therapy to reduce time off work and other indirect costs for patients and their families. Nor do they consider the potential decreases in other healthcare system costs (e.g., hospitalizations) [70,88,89] and socioeconomic costs [70,89].

These potential cost reductions can be significant. For example, from 1980 to 2022, the number of hospital beds required per 1000 Canadians dropped from 6.8 to 2.6 [90]. This happened despite substantial population aging [91]. Several factors, including effective new therapies, have allowed Canada to slash the number of expensive hospital beds.

A QALY value in oncology may be different than in other settings [83]. Some governments apply the same value per QALY across different situations. Others may also consider other factors. These include, first, the level of certainty in judging the drug’s efficacy (although there are differences across jurisdictions on grading and weighting of certainty [92,93,94]). Second, the total potential budget impact of the therapy, including alternative uses of the money (“opportunity costs”). Third is how innovative the medicine is (although there are also different interpretations of what constitutes “innovative” [95,96] and how it should be rewarded [97,98]). Fourth is the potential alternatives for the new therapy [82].

Considering innovation, compared to developing a drug with a novel mechanism of action, there is far less risk involved in developing a drug that works the same way as a proven drug [99]. To encourage innovation, it would be reasonable to pay substantially more for a “first-in-class” drug than for a subsequent drug in the same class unless the subsequent drug offers significant advantages. Approval of multiple drugs with the same target/mechanism of action may add little benefit but can have negative consequences, including disincentivizing innovation [99].

Some governments also weight a QALY value based on the “burden” of the disease, whether the therapy is for a terminal illness, the age of affected individuals, population health inequality issues, etc. [82]. Patients with lethal diseases generally have a short life expectancy. Hence, a therapy for a lethal disease would generally be administered for a relatively short time, and a higher price per QALY would translate into relatively lower lifetime costs. In addition, individuals with severe, life-limiting diseases frequently value their medications differently than those with less severe diseases, and a given level of improvement in health may be of most value to those who are initially sickest [70].

Other calculations have been based on the annual economic productivity of an individual, the pay that would be required to get someone to accept a high-risk job, or the amount one would be willing to pay to reduce risk [82]. The World Health Organization (WHO) has suggested a QALY value of 1–3 times a country’s gross domestic product (GDP) per capita [82].

In some countries, a higher threshold value is applied to a therapy for an “orphan” disease (a serious illness affecting less than 0.05% of the population) [82]. Therapies for orphan diseases are typically much more expensive per QALY than therapies for more common diseases [100]. QALY methods are not well suited to assess the value of therapies for orphan diseases [101]. It may cost as much to develop a drug for an uncommon malignancy subtype or for an orphan disease as for a common disease, but the costs of drug development must then be recouped from a relatively small population when the drug is marketed [102]. In many cases, patients with these diseases may also have a limited life expectancy, meaning that, in addition to only a small number of patients being candidates for these therapies, they may receive them for only a short period of time. Hence, a higher price per patient must be charged for a therapy for an uncommon/orphan disease than for a common disease.

There is generally less research on orphan diseases and uncommon malignancy subtypes. Hence there are fewer available therapy alternatives. Furthermore, it is more difficult to recruit sufficient patients with orphan diseases and uncommon malignancy subtypes to the randomized clinical trials that are needed to increase the degree of certainty of a therapy’s value [5,52]. Requiring the same degree of certainty for orphan diseases and uncommon malignancy subtypes as for common diseases and placing the value per QALY at the same level creates healthcare equity concerns [82]. It means that patients with these uncommon diseases have a much lower probability of being able to access effective new therapies.

Importantly, a therapy for an orphan disease or uncommon malignancy subtype would have little global budget impact since only a few patients would require treatment.

However, in their health technology assessments (HTAs) of new therapies, most governments do not apply different standards to orphan diseases and uncommon malignancy subtypes [103]. There are recurring calls for payors to develop specific funding criteria for this setting that take into consideration why these therapies may be relatively expensive [100]. Both the United States and Europe do offer specific incentives to encourage the development of drugs for orphan diseases [104], but Canada does not.

While the above points could be used to argue that the value of a QALY should be higher for orphan diseases and uncommon malignancy subtypes than for common diseases and for lethal diseases than for nonlethal diseases, some observers might argue that this would not be equitable and that the same value per QALY should be applied in all situations.

However, applying the same value per QALY in all situations would be substantially different from what Canada does in other healthcare and social situations. For example, polio vaccinations, blood transfusions, appendectomies, renal dialysis, heart transplants, or bone marrow transplants can save lives, but there are marked differences in costs for these procedures, and we see no evidence that Canadians question this. Similarly, there are marked differences in the cost to save a life through preventative measures such as car seat belts and airbags, road maintenance, workplace safety regulations, proper disposal of radioactive waste, etc. [105], and we see no evidence that Canadians question this. The amount of income tax paid annually is not the same dollar amount across all Canadians nor the same percent of income, and most Canadians do not question this. Hence, we see no indication that society demands a standard cost per QALY for all situations, nor should it. As outlined above, such an approach would unjustly disadvantage many Canadians with lethal and/or uncommon diseases. From our perspective, it is important to take into consideration not only the cost per QALY but also the broader economic and ethical implications of drug pricing and access.

***Conditional funding models and outcome-based agreements:*** In some countries such as Germany, new therapies may be made available to patients very rapidly after regulatory approval, prior to completion of an HTA or funding negotiation with the drug’s manufacturer [106,107]. Some countries may also negotiate an outcome-based agreement (OBA) with a company [108]. One example of an OBA would be a company providing a drug to a patient free of charge for the first 2–3 months of treatment. If the patient was benefiting and continued the therapy beyond that, the government then funds continued treatment.

***HTA approaches in Canada:*** A description of some of the problems associated with HTA approaches in Canada may inform other jurisdictions. Unlike Germany, England and Wales, France, Italy and Australia, Canada does not have a mechanism to permit publicly-funded early access to innovative oncology therapies prior to HTA assessment [107]. Compounding this is the problem that the HTA process for drugs in Canada is uniquely long and complex [109]. It involves several different bodies and stakeholders [110]. In 2003, Canada established the Common Drug Review under the Canadian Agency for Drugs and Technologies in Health (CADTH) to conduct HTAs on new therapies [110]. (The province of Quebec has maintained its own independent review processes [111]). In 2007, a separate path for HTA of oncology drugs was established, initially through the Joint Oncology Drug Review process. This was subsequently replaced by the pan Canadian Oncology Drug Review (pCODR). It was felt that there were issues specific to oncology that necessitated separate consideration [83,111,112]. However, the pCODR process was transferred back to CADTH in 2014 in order to align pCODR and CADTH practices [110].

From 2011 to 2019, submissions to pCODR with only phase II data were less likely to be approved than with phase III data. Phase II data were particularly likely to be rejected if it was felt that a future phase III trial would be feasible [113]. While there recently have been examples of a few drugs being recommended for approval based on single-arm phase II data [111], there are other instances where this has not been the case [114,115]. The lack of consistency and transparency from pCODR in this context is a source of frustration [109].

From 2011 to 2017, pCODR’s recommendations for conditional approval of an anticancer drug were more likely to be based on unmet needs (with no alternative therapies) rather than cost-effectiveness. The usual maximum recommended cost per QALY was CAD 140,000 (USD 104,000), although recommendations for full approval (rather than conditional approval) took cost-effectiveness into account [116].

In late 2020, CADTH combined CDR and pCODR into a single process (although with a separate expert committee for cancer drugs) [83]. This reduced the maximum value recommended for an oncology drug from CAD 140,000 to CAD 50,000 (USD 37,000) per QALY [83]. Many oncology drugs would require a price reduction of 75% or greater to meet this threshold [83].

The recommendation of CAD 50,000 per QALY was derived from the cost of one year of dialysis in the United States in 1984 [82]. However, this amount has generally not been updated for inflation, and USD 50,000 from 1984 would be worth around USD 150,000 (CAD 203,000) today [117].

As noted previously, the WHO has proposed a QALY value of 1–3 times a country’s GDP per capita [82]. In 2022, Canada’s GDP per capita was USD 54,966 [118]. CADTHs current QALY value of CAD 50,000 (USD 37,000) is only 67% of the lower limit of the WHO’s recommended range. It is also far below the Canadian government’s assessment of the “value of a statistical life-year” (i.e., the government’s willingness to pay to save one life-year by reducing the probability of death). In 2007, this value was CAD 143,000 to 305,000 (USD 106,000 to 206,000) [89]. This currently would be CAD 203,000 to 433,000 (USD 150,000 to $320,000), after correcting for inflation [119].

CADTH has not explained why it is worth so much less to save a Canadian life by enabling access to an effective new drug than for other interventions the Canadian government might consider. Many Canadian oncologists agree that a value of CAD 50,000 per QALY is unacceptably low [109]. It is our view that the CADTH review process has devalued the lives of Canadian cancer patients. We feel that this development has been particularly unfortunate for patients with uncommon malignancy subtypes since they have fewer available therapy options. There are fewer research initiatives that might lead to new therapies, and the large clinical trials required to generate greater “certainty” are often not feasible.

***Pharmaceutical company return on investment (ROI) and public* vs. *industry contribution to new therapies:*** Governments and universities make important contributions to the development of new therapies. However, an estimated 67% of the entire investment in new drugs comes from the pharmaceutical industry [120]. Public funding predominantly supports basic research while industry is the main source of funding for the essential drug discovery and development research that is needed to convert new basic research findings into useful novel therapies [120].

The pharmaceutical industry is more profitable than most other industries, but on average it also invests more in research and development [120,121]. Investing in new drug development is risky, with a high rate of failure. Many new drugs never make it into clinical trials, and of those anticancer drugs that do enter clinical trials, only 3–8% are ultimately approved for sale (although the probability of approval is somewhat higher for “personalized” therapies, with patients selected based on molecular biomarkers) [122]. Investors would not invest in drug development unless the potential ROI was high enough to justify the risks. If pharmaceutical company profits are adjusted for level of risk, they are in line with those of other industries [120].

We believe that the importance of potential ROI cannot be overstated. The average number of new anticancer drugs approved per year has been rising rapidly over recent decades [123]. This progress has been driven by research and the research in turn is driven by investment. This investment, in turn, is driven by the potential ROI. Substantially reducing the potential ROI would decrease investment, research, and progress. Unless government research spending is markedly increased [120], governments alone would be expected to have minimal impact on progress.

***Pharmaceutical company potential actions and reactions:*** While governments are looking for “value”, so are companies. A population generally cannot buy an effective new drug unless the producer is willing to sell it. Following the high costs of required clinical trials, companies then face additional costs in applying for marketing approval [124]. Furthermore, if a company charges a low price early in the life span of a drug, other countries may demand similar low prices. Hence, early sales at a low price could have a negative impact on long-term ROI. Moreover, some drugs may be very expensive to manufacture.

Drugs generally have patent protection for 20 years, but it can take 12–15 years to bring a new drug from discovery to marketing [125]. If a company is to realize an ROI in developing a drug, it would need to charge more for a medication that (for example) requires 15 years to obtain approval (with only 5 remaining years of patent protection) than for a drug that requires only 8 years for approval (with 12 remaining years of patient protection) [2].

Initially, most companies preferentially target markets that offer prospects for the highest probability that they will realize a return on their investment. Countries willing to pay high prices are most attractive, especially if they also have large populations. The United States is usually the first launch country [126] since the US typically has been willing to pay the highest drug prices in the world [1], and they have a population of 340M people. In 2023, it cost a company USD 3.2M to apply to the FDA for marketing approval [127].

After applying to the US FDA, most companies then apply to the European Medicines Agency (EMA) [126]. The EMA approves drugs for more than 450M people living in the 27 countries belonging to the European Union and European Economic Area [128]. In 2023, it cost EUR 345,800 (USD 377,000) to file an EMA application [124].

Following EMA approval, individual countries then negotiate the drug price with the company. Some European countries are small, but some (like Germany) are larger. Some have only been willing to pay relatively low prices for new drugs, but some (like Germany) have been willing to pay relatively high prices [1]. Because a single EMA application covers 27 countries, those with small populations may obtain access earlier than comparable non-European countries. As well, they may pay relatively low prices if there is little added cost for a company to market the drug in that country. Hence, European countries have an advantage that may permit them to access new therapies at prices that are on average lower than for non-European countries [129].

Between 2005 and 2013, companies applied to Health Canada for marketing approval of anticancer drugs an average of 28.4 months after applying to the FDA and 15.5 months after applying to the EMA [126]. Canada has a relatively small population of 40M people, with only about 2.2% of the world market for pharmaceutical sales [130]. In 2023–2024, Health Canada charged CAD 565,465 (USD 418,444) for a new drug application (equivalent to approximately EUR 390,000) [131]. It costs a company more to apply to Canada than to Europe, despite Canada having a much smaller population.

Cancer is the leading cause of death in Canada [132]. In 2021, the total economic burden of cancer in Canada was more than CAD 26B (USD 19B), yet patented oncology drugs accounted for only 1.3% of national health expenditures [89]. It is anticipated that this will increase. Pharmaceutical spending accounted for 18.2% of Canadian healthcare spending in 2010 but had fallen to 14.5% by 2022 [133]. Similarly, the amount spent on patented medicines in Canada was 0.75% of GDP in 2002, peaked at 0.83% in 2009, then fell to 0.76% by 2021 [1]. However, sales of oncology drugs increased from 8.4% of patented medicine costs in 2012 to 23.9% in 2021 as the number of patented cancer medicines went from 56 to 113 and sales went from CAD 1.379B (USD 1.02B) in 2012 to CAD 4.172B (USD 3.09B) in 2021 [1]. With the increasing availability of effective new drugs, it is anticipated that this could rise substantially over the next few years and that this will put increasing pressure on the healthcare system.

Canada has previously been willing to pay relatively high prices for drugs [1], but there has been a recent move away from this. As noted previously, CADTH has set an ICER threshold of CAD 50,000 (USD 37,000) per QALY gained. Moreover, Canada’s Patented Medicine Prices Review Board (PMPRB) also introduced new regulations that would reduce the maximum price that could be charged for a drug in Canada by about 17% to the median of prices charged in 11 comparator countries [134].

We agree with assessments by others [129]. Our unpublished analysis of data presented by PMPRB [135] indicates that the relative amount that a country is willing to pay for drugs correlates strongly with the proportion of new drugs that the country can access (*p* < 0.0006 for EMA countries and *p* < 0.007 for non-EMA countries). Extrapolating from our analysis, we estimate that PMPRB’s proposal to reduce maximum Canadian drug prices by 17% will decrease the number of new drugs available to Canadians from about 48% of the global total to about 35%. Moreover, access to those drugs that are launched in Canada would be delayed [129].

Potentially, these delays could be partially mitigated by new initiatives such as Project Orbis [136], or similar approaches to tie rapid Canadian approval to US or EMA approval [89]. In Project Orbis, a company’s application for funding approval in Canada is integrated with the application to the FDA and five other nations. This has the potential to both accelerate and increase applications to Canada. Overall, Health Canada is currently working on regulatory changes to take lessons learned from the COVID-19 pandemic to accelerate Canadian marketing approval of effective new agents [137]. We applaud Health Canada for this initiative.

Canada’s HTA and price negotiating processes following Health Canada’s approval of a drug are very inefficient [109]. Public funding for drugs approved from 2012 to 2021 did not occur until an average of 2 years after Health Canada approval, which is about twice the average of peer countries in the Organization for Economic Cooperation and Development (OECD) [138]. In 2021, it took an average of 2.5 years from Health Canada’s approval of a drug to public funding, and in 2022, it took an average of 2.3 years [139].

Of 29 reporting OECD countries, Canada ranked 22nd in public funding of 460 new medications launched globally from 2012 to 2021 (18% for Canada vs. an OECD average of 28%) [138]. Canada ranked far behind the US and European Union in publicly funding new anticancer drugs that were approved between 2016 and 2020 [89]. On average, the delay is 3.7 years after public funding in the US and 2.9 years after public funding in Europe. The majority of the delay is due to the time taken for HTA assessment, negotiation of public funding following regulatory approval and provincial listing of a drug [89]. Such impaired access to effective new cancer therapies can translate into thousands of Canadian life-years lost [140].

When new anticancer drugs are approved for funding in Canada, this funding is fragmented. It varies from one region of the country to another [139] and differs for oral medications compared to intravenous medications [141].

Canada has the highest rate of increase in medical assistance in dying (MAID, physician-assisted suicide) in the world [142] (and MAID has the potential to save the Canadian healthcare system up to CAD 140M (USD 104M) annually [143]). It is unfortunate that Canada cannot do as well in offering patients hope and relief from suffering by making effective new cancer therapies available to them. Canada is leading in the wrong category.

## 4. Endpoints: Problems with ICERs

There are additional problems intrinsic to calculating, interpreting, and applying ICERs, as outlined below. Some potential solutions are listed in Table 1.

***Crossover:*** Overall survival (OS) gain is regarded as the “gold standard” metric for assessing the efficacy and value of a new therapy [144]. OS gains in randomized clinical trials (RCTs) are often used in calculating ICERs. Many trials allow crossover from the control arm to the experimental arm. If patients in the control arm live longer because of this crossover, then the OS difference between the experimental arm and the control arm is artificially reduced and less likely to achieve statistical significance [55]. Trials testing more effective agents are more likely to have high crossover [55]. Consequently, OS ICERs undervalue effective agents if crossover is permitted. Because of this, we feel that OS is clearly an unreliable clinical trial endpoint if there is a high level of crossover. However, crossover can be good for patients, and prohibiting crossover to an agent that appears to be effective would be unethical [55]. If a trial has more than a low level of crossover, an endpoint other than uncorrected OS gain is needed to determine a therapy’s value. We believe that progress is impeded unnecessarily when useful therapies are erroneously discarded or undervalued due to trials with false-negative OS endpoints.

***Differences in efficacy estimate depending on OS outcome assessed:*** Some new therapies have had a major OS impact, including, for example, new immune checkpoint inhibitors in metastatic non-small cell lung cancer [145]. However, not all OS measures are equal, and different OS measures can be used to determine the relative benefit of a new therapy. These include median OS, mean OS, 1-year or 3-year OS rate, and number needed to treat to avoid 1 death at 1 year. Without taking these differences into account, the relative clinical OS value can be inconsistent across different therapies and tumor types [146,147]. A new therapy might have minimal impact on median OS or the OS hazard ratio but might result in a meaningfully higher proportion of patients still alive at 3–5 years, as is often seen with immune checkpoint inhibitors. For individual therapies, it is often unclear which aspect of the OS outcome is used to calculate the ICER. In their “Value Framework”, the American Society of Clinical Oncology (ASCO) recommends assigning bonus points to a therapy that substantially improves late OS [77]. Unlike the average physician surveyed, the average cancer patient placed a higher value on at least a small possibility of long-term survival than on a more certain improvement in median survival [148]. Simply put, patients with a terminal cancer diagnosis, but not their physicians, prefer a “risky gamble” to a “safe bet”. Patients with lethal diseases may assign a high value to hope [70].

***Inaccurate median OS due to midpoint deviations of Kaplan–Meier curves:*** ICERs also may be inaccurate if they rely on differences in median OS between experimental arm and control patients. If the Kaplan–Meier curves of the two groups deviate at their midpoints, this can result in over- or underestimation of the OS difference between the two arms. Differences in OS half-lives give a more accurate estimate of the OS gain since half-lives are less impacted by Kaplan–Meier curve deviations [55]. (The online appendix of a recent open-access publication [149] includes a step-by-step tutorial on the use of readily available tools to easily calculate half-lives from published survival curves).

**Figure 1:**
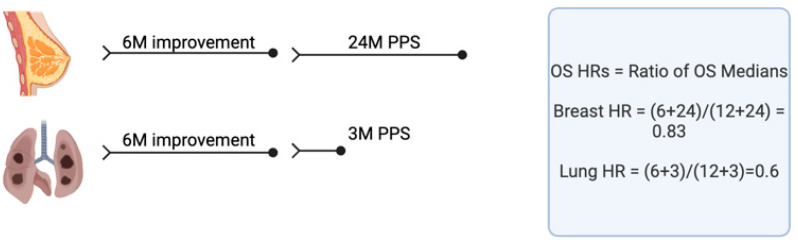
For a drug with the same absolute impact on median OS in two different malignancies (e.g., breast cancer and lung cancer), differences in post-progression survival (PPS) would mean that the therapy had a greater impact on the OS hazard ratio (HR) for lung cancer than for breast cancer.

***Hazard ratios and impact of post-progression survival (PPS):*** Hazard ratios also may be used instead of OS differences in judging a drug’s value. Both crossover and PPS can impact OS hazard ratios. For example, let us consider a drug that increases median PFS from 6 months to 12 months in both breast cancer and lung cancer (Figure 1). However, in breast cancer, there are additional therapies that can be effective after progression, and median PPS is 24 months for both the control arm and the experimental arm. Since OS hazard ratios are roughly equivalent to the ratio of the OS medians of the study arms [55], the OS hazard ratio would approximate (6 + 24)/(12 + 24) = 0.83. Based on this hazard ratio, the impact of the new therapy would be judged to be only modest. However, in lung cancer, there is no effective subsequent therapy in this example, and median PPS is only 3 months on both arms of the trial. Here, the OS hazard ratio would be (6 + 3)/(12 + 3) = 0.6, and the new therapy would be considered to be effective. The absolute impact of the new therapy on OS would be the same in both breast cancer and lung cancer, but the hazard ratio would suggest a greater benefit in lung cancer than in breast cancer.

***Hazard ratios and relative* vs. *absolute gain:*** As noted, hazard ratios may not reflect the absolute gain with a new therapy. If a new therapy increased the median OS from 1 week to 2 weeks, the hazard ratio would approximate 0.5. The hazard ratio would suggest that this therapy is “effective”. In another setting, if a new therapy increased the median OS from 10 years to 11 years, the OS hazard ratio would approximate 0.91, and the new therapy might be deemed “ineffective”. The absolute gain in OS may be taken into consideration in judging whether a trial is “positive”, but it may not be considered in calculating the ICER. As noted earlier, misperceptions that a drug’s value is higher than it really is might result in paying too much for it [4], but misperceptions that a drug is less valuable than it actually is can also be problematic. Excessively negative misperceptions based on flawed interpretations of clinical trial data may result in useful drugs being discarded. We feel that some authors [144,150,151] may undervalue the benefits of effective new therapies by inadequately considering the impact of crossover and long post-progression survival on OS absolute gains and hazard ratios.

***p-value:*** Conventionally, a *p*-value < 0.05 may be used as a strict threshold to determine the efficacy of a new therapy. However, the use of a *p*-value of 0.05 as an indicator of statistical significance is an arbitrary convention [152], with no intrinsic biological or clinical meaning [153]. Many statisticians now question the reliability of *p*-values in decision-making [153,154,155,156,157,158].

Current practice among many clinicians, regulators and payers assumes that there is a difference between a *p*-value of 0.051 and a value of 0.049 [4]. However, from the point of view of probability, we believe that any reasonable person would conclude that they both mean the same thing. From our perspective, using cut points in assessing *p*-values is irrational.

Importantly, *p*-values are a measure of certainty and are only an indirect measure of effect size [153,154]. The precision (and hence the *p*-value) is greatly impacted by the number of patients on the trial, and a large trial can have an impressive *p*-value despite only a small gain. For example, a cardiology study comparing tPA to streptokinase/heparin after myocardial infarct included 41,021 patients and demonstrated *p* < 0.001 favoring tPA, despite having an absolute improvement in survival of only 1% [159].

Consider a hypothetical trial with 100 patients in which the OS is increased from 1 year to 2 years, but because of low patient numbers and low statistical power, the *p*-value is 0.06. Based on *p*-values, this therapy which gives a 1-year improvement in OS would be considered marginally effective and would probably be discarded despite a high probability that its benefit is real. Conversely, the cardiology therapy that gives only a 1% OS improvement would not be discarded.

In studies with multiple comparisons, there is an increased probability that one of the comparisons might reach *p* < 0.05 based on chance alone [160]. Hence, if there are going to be multiple comparisons, the alpha value is “split”, and the trial requires a much smaller *p*-value for statistical significance. Hence, if the required OS *p*-value is set at 0.01 (for example), and the study instead detects an OS *p*-value of 0.015, the therapy will be discarded. For example, in the assessment of pembrolizumab in extensive small-cell lung cancer, the required OS *p*-value was 0.0128 based on the performance of multiple comparisons. OS failed to achieve statistical significance since the observed *p*-value was instead 0.0164 [161], and in the US, pembrolizumab has recently been withdrawn as a potential therapy for extensive small-cell lung cancer.

With that *p*-value, there still might be a high probability that the new therapy is of benefit. Moreover, it is likely that each of the multiple comparisons will have detected a favorable *p*-value. It is the use of *p*-value cut points that makes this an issue: if you just cross the line once you win, and if you try many times, you are more likely to cross the line at least once. If you only take into consideration the probability of benefit, and not whether you have crossed a hard *p*-value line, then this problem goes away. You can then perform as many comparisons as you want, and fewer effective therapies are inappropriately discarded due to the multiple comparisons trap.

In contrast to *p*-value cut points, Bayesian approaches can have advantages by exploiting a priori available information and by giving a probability that a new therapy is beneficial [162]. In addition, with Bayesian analyses, one does not have to correct for multiple comparisons [162].

***PFS:*** Because of the impact of crossover and long post-progression survival (PPS) on OS outcomes, there has been an increasing reliance on PFS gain as a primary trial endpoint [163]. This can create challenges in calculating a therapy’s “value”. Because PPS has little impact on PFS outcomes, PFS hazard ratios and *p*-values may be very favorable despite the absolute gain in PFS being very small [55]. Also, while PFS hazard ratios correlate highly with OS hazard ratios in many oncology settings, they are poor predictors of OS hazard ratios. This is due to the impact of crossover and PPS on the OS hazard ratio [55,164]. On average, PFS hazard ratios are more favorable than OS hazard ratios and are more likely to be statistically significant [55,164].

Gain in median PFS is also a relatively weak predictor of OS gain [55]. However, gain in PFS half-life is a good predictor of OS half-life gain [55]. Across most solid tumors and therapy types (with the exception of metastatic prostate cancer or use of toxic, very high dose therapies), a PFS gain ≥1.5 months predicts an OS gain of ≥2 months, with a positive predictive value of 90% and a negative predictive value of 86% [55,165]. PFS gains higher than this predict even higher OS gains [55]. In the presence of a high crossover rate or long post-progression survival, PFS half-life gain is a more reliable indicator of true OS benefit than is an OS *p*-value or hazard ratio [55], irrespective of whether one regards PFS gain as being of value in its own right.

While the PFS half-life gain predicts if an OS gain will be greater than a certain minimal amount, it does not predict with certainty how high it will be. In some instances, the OS gain may be much greater than the PFS gain [55,165]. Hence, the PFS gain may tell us that the new therapy is “of value”, but it may underestimate how much value, if OS gain is our measure of value.

In their “Value Framework”, ASCO proposes using PFS in calculating value if data on OS are not available, but they discount the PFS benefit by multiplying the benefit by 0.8 [77]. Since PFS hazard ratios are generally superior to OS hazard ratios [55], this appears to be a reasonable approach. However, PFS half-life gain is a better predictor of OS gain than the PFS hazard ratio [55]. When PFS gain is ≥1.5 months and statistically significant, the absolute gain in OS half-life is generally greater than the absolute gain in PFS half-life [55,165]. Since the PFS half-life gain is likely to underestimate the OS gain, if differences in PFS half-lives are used instead of PFS hazard ratios, we believe that the PFS gain should be judged to be of increased value, rather than being discounted by multiplying by 0.8.

***Response:*** Some single-agent therapies are also approved based on high response rates in non-randomized trials. This is a reliable indicator of drug efficacy [166]. Response rates correlate strongly with survival [167,168] and with eventual regulatory approval of a drug [169]. The FDA’s “breakthrough drug” designation permits rapid approval of effective new agents based on high response rates in phase I/II trials [46], and these drugs have generally stood the test of time [170].

In many countries, a high phase I/II response rate is used as a basis for drug funding. Canada is inconsistent on this. For example, both tepotinib [171] and capmatinib [172] are associated with high response rates in non-small cell lung cancers with an *MET* exon 14 skip. However, neither is publicly funded for most Canadians.

“Response” is defined as a ≥30% reduction in diameter of measurable tumors, lasting at least 4 weeks [173]. A 30% reduction in tumor diameter is required since tumor measurements are imprecise. For example, up to 14% of patients may appear to have a >10% change in tumor diameter solely due to measurement imprecision [174].

However, it is uncommon for tumors to have an apparent ≥30% reduction in tumor diameter due to measurement imprecision [174]. In an assessment of placebo or best supportive care arms of 25 clinical trials, the median RECIST response rate was 1% [166]. Overall, 3 of the 25 trials had a response rate of 3%, one had a response rate of 4%, but none had a higher response rate than 4%, and no responses were noted in 11 of the trials [166]. Overall, tumor regression >30% usually means that the tumor regression is “real”. Even if the observed response rate is relatively low, the lower limit of the 95% confidence intervals for the response rate may often be greater than 0%.

In their “Value Framework”, ASCO lays out criteria for using response rate data for single-arm trials [77]. In rating the benefit, they “discount” the response as an outcome by multiplying the response rate benefit by 0.7 [77]. The rationale for doing this is unclear. New therapies with high response rates may be of substantial benefit to patients, provided the responses are sustained for several months or longer. Response and improvement in symptoms, PFS and OS are all driven by the same process: killing a tumor cell using a therapy. This is why they correlate strongly with each other.

For some drugs, responses may last for many months or longer, while for others, they may last only weeks to a very few months. Our perspective is that a few responses lasting a long time might be regarded as being more valuable than a higher response rate, but with a short response duration. It would be worthwhile exploring the development of a composite endpoint that incorporates both response rate and duration.

Overall, for single agents, a high response rate in a non-randomized trial is an indicator of drug efficacy [46,166,167,168,169,170]. There is less clarity with drug combinations. Unless the lower limit of 95% confidence intervals for the response rate for the combination is very high, it may not be high enough to exceed the upper limit of the 95% confidence intervals for a single-agent therapy. Hence, RCTs may not be needed to establish the value of a single agent, particularly if the response rate is high. However, in many cases, they will be needed to confirm the value of a combination compared to a single agent unless the response rate for the combination is very high.

***Quality of life (QOL)/symptom improvement:*** QOL may also be incorporated into the calculation of ICERs. QOL is very important, but the measurement of QOL is much less precise than the measurement of OS, PFS or response. In addition to being imprecise, it can vary substantially over a short period of time, there is disagreement on how these variations over time should be scored, and potential bias remains an issue in QOL assessments [175]. Furthermore, while several different factors are explored in QOL assessments, the range of factors is nevertheless limited [176]. Overall, QOL assessments continue to have conceptual and methodological challenges [177].

In cancer patients, QOL is predominantly directly or indirectly dependent on cancer symptoms and treatment toxicity, although other factors also contribute. Fatigue, as an example, might have a major impact on QOL early after a cycle of chemotherapy, but far less impact just a few days later [4].

Cancer symptoms typically improve if the cancer shrinks with treatment or worsen if the cancer grows. However, some long-term structural alterations (e.g., a pathological fracture or a trapped lung) may result in symptoms persisting despite tumor regression [4]. While a new effective therapy may be superior to an older therapy in improving several cancer symptoms, it may not substantially improve symptoms related to long-term structural alterations. QOL assessment is a blunt instrument that does not differentiate well between symptoms that could or could not be improved by making a cancer shrink. Hence, it may tend to underestimate therapy benefits.

If two therapies induce the same degree of tumor regression, we assume that they would result in generally the same degree of improvement in cancer symptoms. There are likely to be few exceptions to this. While assessment of objective response can have some degree of error [166,173,174], we expect it to be substantially more precise than subjective assessments such as QOL [178] or symptom improvement. In deciding on the value of a new therapy, our perspective is that response rate and duration are likely to predict the probability of symptomatic improvement.

With respect to drug toxicity, some side effects (e.g., neutropenia) may be asymptomatic, with far less impact on patient QOL than, for example, fatigue [4]. Furthermore, many drug side effects may decrease substantially with dose reduction and are therefore modifiable. On the other hand, dose–response curves for solid tumors are relatively flat for most therapies. Hence, reducing drug doses may decrease toxicity, thereby improving QOL without substantially reducing efficacy [179].

However, many clinical trials limit the extent of permitted dose reductions. This can limit the ability to reduce toxicity and adversely impacts QOL compared to what might be possible in a real-world situation [179]. While toxicity is a reality with most anticancer therapies, it is the job of an experienced oncologist to try to limit this toxicity to the extent possible, while maintaining therapy efficacy.

Research focusing on identifying subgroups of patients at risk of overtreatment and treatment de-escalation strategies can also help improve toxicity.

While the impact of toxicity on QOL may be incorporated into a QALY assessment, we feel that this may ultimately add little value when deciding how much to pay for a new medication. If two therapies with similar efficacy have the same price, but therapy A is less toxic than therapy B, then oncologists and patients will preferentially choose therapy A. Therapy A will then be rewarded for its reduced toxicity based on sales volumes, with little gained by trying to determine a price differential. In fact, the Canadian healthcare system has examples of a less toxic option not being publicly funded since a more toxic option is less expensive. An example is afatinib vs. osimertinib in non-small-cell lung cancer patients with uncommon *EGFR* mutations (mutations other than exon 19 deletion, L858R mutation or T790M mutation). Paying less for more toxic therapies can impede access to better-tolerated or more convenient alternatives.

However, there may be a good reason for also wanting access to the more toxic Therapy B after Therapy A. For example, in the treatment of metastatic NSCLC with an *ALK* fusion gene, cross-study comparisons suggest that lorlatinib may be slightly more effective than alectinib. However, alectinib is generally well tolerated, while lorlatinib is more toxic. Hence, an oncologist may recommend initial therapy with the slightly less effective but less toxic drug alectinib, with the option of considering a switch to lorlatinib when alectinib no longer works.

The bottom line is that QOL is very important, but, from our perspective, QOL data must be interpreted cautiously. Do data on improvement in cancer symptoms add anything meaningful over and above OS, PFS and response data? Do QOL data related to therapy toxicity reflect the impact of steps an experienced oncologist might reasonably have taken to reduce toxicity outside of a clinical trial setting? How do patients weigh (for example) improvement in severe cancer-related cough vs. therapy-associated fatigue? It is important to keep in mind that the most common cause of loss of health-related quality of life in this context is progressive cancer.

Many patients accept grade 1–2 therapy toxicity in return for improvement in cancer symptoms and prolonged control of their cancer. Slevin et al. reported that the average patient would accept a highly toxic therapy in return for just a 10% possibility that their cancer symptoms would improve, and would accept a moderately toxic therapy in return for just a 1% chance of cancer symptom improvement [68]. Patients dealing with cancer were much more willing to accept therapy toxicity in return for improvement in symptoms than were healthcare providers or members of the general public [68]. These, and other findings reveal the importance of the patient being heard more clearly in setting priorities in clinical research [180].

***Efficacy in subpopulations:*** A new therapy may work much better than a standard therapy in one subpopulation but not in another. The relative lack of efficacy in one subpopulation might be enough to result in a negative RCT when compared to a standard therapy [166]. To discard the new therapy based on this negative RCT would deprive a subpopulation of patients of a therapy that is of substantial value to them.

There are two clues that a drug may be working well in a distinct subpopulation. The first is the ability of the new therapy to yield responses in some patients who are clearly resistant to the standard therapy. The second is the shape of the log–linear PFS curve [181]. If there is an inflection point to the right on the log–linear curve and if the curve fits a 2-phase decay model on exponential decay nonlinear regression analysis, this suggests two distinct subpopulations with differing rates of progression. (See the online appendix of a recent open-access publication [149] for a tutorial on how to easily assess this).

For example, across a range of malignancies, immune checkpoint inhibitors yield responses in 10–20% of patients who are resistant to chemotherapy. A majority of PFS curves for single-agent immune checkpoint inhibitors show 2-phase decay. For one subpopulation, PFS may be shorter than with standard therapy comparators, while PFS is much longer than with the standard therapy for the other, smaller subpopulation comprising 10–20% of the total population [181]. This pattern of efficacy alone tells us that the therapy is of potential value, even if OS outcomes do not achieve statistical significance.

***Real World Evidence (RWE):*** Post-marketing RWE may also be used to assess therapy efficacy. Some authors have proposed using RWE in making decisions on drug funding. This may be particularly useful in uncommon malignancies where it is difficult to generate clinical trial data. An example is the use of RWE in the FDA approval of Palbociclib for male breast cancer [182].

However, RWE cannot be generated unless the drug is in common use, and it cannot be in common use without funding. Consequently, there are limitations in using Canadian RWE to make decisions about funding.

One way around this would be to make the drug available to patients very rapidly after it has attained regulatory approval and prior to HTA assessment and formal price negotiations. RWE can then be collected during the period of HTA assessment. This approach is currently taken by Germany and some other countries [106,107].

While RWE data can be associated with substantial uncertainty [94], we feel that they might give a more accurate assessment of true drug value than the current approach of using assessments based on clinical trial data alone.

Another potential use for RWE is as an “external” control arm for trials of new drugs [183]. This could substantially reduce clinical research costs.

***Sequencing and comparisons of agents with differing mechanisms of action:*** ICERs are used to determine if a new drug is superior to an older drug and can replace it. However, if the drugs work by different mechanisms, and both the old and the new drug can cause tumor regression in at least some patients, our perspective is that the optimal approach generally would be to follow one drug by the other, rather than replacing one by the other. Across therapies and tumor types, previously treated malignancies are generally less responsive than treatment-naïve malignancies. Despite this, most therapies that are effective as first-line therapies also offer meaningful benefits in previously treated patients. The average patient benefits most from receiving one and then the other, not by receiving one instead of the other. This is an essential concept obvious to most oncologists and patients.

## 5. Discussion

Governments are facing a difficult conundrum. Therapies keep improving while concurrently becoming much more expensive. The prices of these therapies are not affordable for most patients. They also may rapidly reach the point at which government healthcare systems cannot afford them either. In addition to the financial burden of these therapies, there is the inconvenience and toxicity associated with taking the therapy, but patients regularly make the decision to accept the inconvenience and toxicity. The major hurdle we are facing is the financial hurdle.

In Canada, while the overall cost of pharmaceuticals as a percent of healthcare spending is not rising, and while the cost of patented drugs as a percent of GDP is not increasing, oncology drug sales as a percent of patented medicine sales are increasing rapidly [1,133]. The good news is that these new medications have resulted in substantial improvements in outcomes for cancer patients [4,184]. The bad news is that with the rate of development of these effective but expensive new medications, we do not believe that we will be able to continue to afford them unless something changes [4,15,44].

We have only one of two choices. We can bring down drug prices by taking the major steps required to substantially reduce the exorbitant costs of new drug development. Or we can accept a marked slowing of research and progress. As demonstrated by AIDS therapies and COVID-19 vaccines, governments have it in their power to fix this but have yet to take any meaningful action.

There may be little urgency in developing therapies for nonlethal diseases. However, this is not the case when it comes to lethal diseases, where the situation is urgent. We must radically transform our regulatory approaches to reflect this [4,15,44].

Canada is following a disturbing pattern. Not only has Canada fallen behind OECD peers in public funding for effective new cancer therapies [138], but it also lags behind the majority of other OECD countries in other healthcare metrics, including numbers of physicians [185], hospital beds [186], CT/MRI/PET scanners [185,187,188] and radiotherapy machines [186] per million population. Canada’s economic health depends on its ability to maintain a healthy population and to attract and retain skilled immigrants. Canada’s ability to do this is seriously compromised if it has inadequate healthcare infrastructure. Canada’s healthcare is not just an expense: it is an essential investment. It is our belief that Canada is not investing enough.

Those who negotiate on our behalf to purchase new drugs have a very difficult task. However, trying to reduce it to a simple QALY-based equation is clearly not the answer. We feel that such equations make several invalid assumptions, are not patient-centric, and fail to consider the costs of drug development and production. They risk worsening the inequity faced by those with less common and more serious medical conditions, denying patients access to effective new therapies that can prolong their survival and offer hope and relief from suffering. The system is clearly broken. It is time to fix it!

The quest has been to find a single metric that defines the value of a new anticancer therapy. This would greatly simplify the decision of how much to pay for the therapy. However, the bottom line is that no such gold standard metric exists. Any metric that we might choose will have intrinsic limitations, and trying to designate one as being our gold standard will have a high potential to result in very flawed decisions.

We must weigh all the data together, including OS half-life gain, OS hazard ratio, the proportion of patients with prolonged OS, PFS gain (if crossover, long post-progression survival or short follow-up distort OS outcomes), level of certainty in the outcome, Bayesian “prior probabilities” that might support our view on the therapy, impact in distinct subpopulations and in disease that is resistant to other therapies, single agent response rate and duration of response, the proportion of patients with prolonged responses, the improvement in response and duration of response when the new agent is added to a standard therapy, therapy toxicity and convenience and ease of administration, availability of alternatives, impact on other components of the healthcare system (e.g., hospital beds and emergency departments), etc. Then, once we have made our decision and negotiated a price, this must be regularly renegotiated as we assess real-world evidence derived from patients who have received the therapy.

In negotiating the price, we must also be willing to pay a “premium” for a therapy that is beneficial in a lethal or orphan disease.

While doing this, we must work to correct factors that impede competition, and we must do everything feasible to make new drug development cheaper and faster. If it is cheaper and faster, that will increase the options available to us, and help bring down therapy prices while speeding the development of even better therapies in the future. The rate of progress against cancer has been unprecedented. By far the biggest threat to sustained progress is the rapidly rising cost of new drug development and the potential inability of governments to afford the new therapies that are discovered [4,15,44]. It will be a pyrrhic victory if the government attempts to cut drug prices at the cost of a marked slowing of research and progress [9].

## Figures and Tables

**Table 1:** Recommendations.

Issue	Recommendations
**Issues with Clinical Research Oversight:**	
There are too many impediments to rapid progress against lethal diseases.	We need revised oversight of clinical research for lethal diseases that will: Move much faster. Accept higher risk. Involve progress-centered regulation. Include rationalized privacy regulation that is much less obstructive.
Preclinical toxicology requirements are excessive.	We only need LD_10_ in rodents (i.e., the drug dose that kills 10% of rodents).
Study review and activation processes are too slow and inefficient.	We need: Review by a single international accredited IRB, not dozens of local IRBs. Concurrent/parallel—not sequential—protocol-review procedures. Time-limited approval processes. Standardized, preapproved protocol templates. “Just-in-time” trial approval processes for uncommon diseases (an institution can download/rapidly activate a trial already approved elsewhere by an accredited IRB).
Trial eligibility criteria are irrationally rigid.	We need to: Permit trial principal investigator to approve participation of “borderline” patients. Eliminate exclusion based on: Data cut points (e.g., creatinine above vs. below specified value). Metastatic sites (e.g., brain metastases). Prior malignancy (unless it is uncontrolled).
Requiring an accredited test for molecular screening/selection for a clinical trial can greatly delay trial activation and escalate costs (if test is not already active and accredited).	Permit screening using a research lab test, with later refinement/accreditation of the test if it is determined to predict therapy benefit.
Regulatory burden of running a trial is excessive.	The trial principal investigator can unilaterally change trial details as needed to improve trial feasibility and efficiency, without requiring IRB amendment approval (e.g., lowering therapy doses, dose intensity or treatment frequency, changing the frequency of follow up testing, broadening or tightening trial eligibility criteria, etc.) Long-term follow-up on study participants permitted without requiring annual trial reapproval.
Trial data collection requirements are expensive, burdensome, wasteful, and excessive.	Rationalize data collection.
We cannot change clinical research regulation locally since it is “harmonized” internationally.	We need rapid international collaboration on making research regulation much more efficient, much less costly, and much more progress-centered for lethal diseases.
**General issues with delayed access to new therapies in Canada:**	
Companies delay applying to Health Canada for drug marketing approval.	Generally, Canada should approve a drug for a lethal disease as soon as the US FDA or the EMA approves it (e.g., through Project Orbis) without further extensive review.
Public funding of new drugs takes much too long in Canada.	Canada needs: A government culture committed to maximizing rapid access to effective new therapies for Canadians with lethal diseases. A conditional funding model or outcome-based agreement that permits immediate access for all Canadian cancer patients to all new cancer drugs relevant to their illness. A Canadian accelerated approval pathway for promising non-randomized data (e.g., response rate from phase I-II data). Outcome-based agreements could be particularly helpful for therapies approved in this manner. HTAs that begin as soon as Health Canada or Project Orbis begins the assessment of a drug. Initiation of negotiation with companies on drug price and access as soon as Health Canada or Project Orbis begins the assessment of a drug. Government recognition that an effective new therapy for metastatic cancers or other lethal diseases will generally be given in sequence with (before or after) established therapies rather than instead of established therapies. To exclude drugs for cancer and other lethal diseases from PMPRB price regulations (as previously done for COVID-19 vaccines [53]).
**Issues with ICER and QALY calculation and value in Canada**	
General factors that are considered in calculating an ICER and/or the value of a QALY for a new therapy need to be updated.	For ICER and QALY calculations for therapies for lethal diseases, we need: Full transparency about how they are calculated. Adjustment of QALY values based on inflation. Linking of QALY value to: 1–3 times Canada’s per capita GDP (as recommended by WHO). The Canadian government’s current “Value for a Statistical Life-Year”. How much the therapy saves other parts of the healthcare system (e.g., in decreased hospitalizations) and positively impacts the overall economy (e.g., by allowing patients to return to work). The degree of innovation/novelty. How it compares to other therapies with the same mechanism of action (as potentially assessed using simple, inexpensive trial approaches such as the REaCT methodology [54]). Rarity of the disease and the associated unmet need.
CADTH assigns the same value for a QALY to all disease types.	For lethal diseases (and particularly for uncommon malignancy subtypes and rare or orphan diseases), the upper value assigned per QALY should be at the higher end of either 3 times Canada’s GDP per capita or the upper limit of the Canadian government’s “Value per Statistical Life-Year”.
Different trial endpoints are not adequately considered in calculating QALYs, ICERs and therapy efficacy and value.	In calculating QALYs, ICERs and therapy efficacy and value, we should consider: Correcting for the impact of crossover on control arm OS (e.g., by censoring control arm patients at the time they crossover, or by using historical controls or Real World Evidence for the control arm). Using gain in OS half-life [55] rather than OS hazard ratio or gain in OS median. Using a composite score that incorporates both gain in OS half-life [55] and gain in proportion of patients surviving at a relevant late time point (e.g., 3 or 5 years) if there are nonproportional hazards. Accepting that a new therapy is effective if either the PFS half-life gain or the OS gain exceeds a relevant threshold. For example, since a PFS gain ≥1.5 months predicts a high probability of an OS gain ≥2 months if OS is undistorted by crossover [55], accept the therapy as effective if the PFS half-life gain is ≥1.5 months with *p* < 0.05 or the OS half-life gain is ≥2 months with *p* < 0.05. Moving away from statistical cut points (e.g., the use of *p* < 0.05 to determine “significance”) to (a) using all available evidence in judging efficacy and to (b) using Bayesian statistical approaches (which do not rely on *p*-value cut points, which can include a variety of different lines of evidence in the form of prior expectations, and which do not require a correction of the *p*-value for multiple comparisons). In a situation where OS data are unavailable or are unreliable due to high crossover, using PFS half-life gain and multiplying it by 1.2–1.3 to obtain an estimate of OS gain (since a PFS half-life gain ≥1.5 months predicts a high probability of an OS gain ≥2 months, and a PFS half-life gain ≥5 months predicts an OS gain ≥6 months [55]). Developing a value assessment that incorporates both response rate and response duration. Using life-years gained and not quality-adjusted life-years gained (QALYs) in calculating ICERs and value since QOL measurements are very imprecise and can vary over short periods of time. Funding therapies that are of value in resistant subpopulations, even if they are not superior to standard therapies in RCTs.
